# Safety and efficacy of antibiotic-impregnated absorbable calcium sulfate beads (Stimulan) in cranioplasty

**DOI:** 10.1093/jscr/rjae468

**Published:** 2024-07-23

**Authors:** Alexander R Evans, Marianne E Kimmell, Abdurrahman F Kharbat, Hakeem J Shakir

**Affiliations:** Department of Neurosurgery, University of Oklahoma, Oklahoma City, OK 73104, United States; Department of Neurosurgery, University of Oklahoma, Oklahoma City, OK 73104, United States; Department of Neurosurgery, University of Oklahoma, Oklahoma City, OK 73104, United States; Department of Neurosurgery, University of Oklahoma, Oklahoma City, OK 73104, United States

**Keywords:** antibiotic, calcium sulfate beads, cranioplasty, Stimulan

## Abstract

Cranioplasty is a common neurosurgical procedure that follows hemicraniectomy in the setting of neoplasm resection or increased intracranial pressure. Although standardized practices aim at minimizing infection risk, infection of the surgical site has been reported in 6.6%–8.4% of patients. In this work, we document the novel use of synthetic dissolvable antibiotic-impregnated calcium sulfate beads (STIMULANⓇ Rapid Cure, Biocomposites Ltd, Wilmington, NC, USA) in five cases of cranioplasty at our institution. Four patients experienced wound healing as expected with no complications related to the use of Stimulan beads. One patient’s clinical course was complicated by pseudomeningocele with superficial wound infection occurring 74 days following cranioplasty. Of note, this patient had suffered an avulsion injury and subgaleal hematoma of the scalp ipsilateral to the cranial incision, predisposing to infection due to incompetent scalp vasculature. No complications could be directly attributed to the use of STIMULANⓇ beads.

## Introduction

Cranioplasty is a neurosurgical procedure that commonly follows hemicraniectomy in the setting of neoplasm resection or increased intracranial pressure. Although largely utilized for cosmesis and psychosocial benefit, restoration of cranial architecture has also been associated with the improvement of cerebral blood flow and cerebrospinal fluid dynamics [1]. Standard practice includes measures to minimize postoperative complication rates, often including application of antibiotic powder extradurally prior to closure; however, infection of the surgical site occurs in 6.6%–8.4% of patients, despite the administration of broad spectrum antibiotics [2, 3]. Given the notable incidence of infection, innovative techniques have emerged in efforts to minimize perioperative complication rates.

Within the setting of bone and joint infections, antibiotic-impregnated synthetic calcium sulfate beads have become a popular option in combating sepsis following orthopedic procedures [4–16]. Here, we report the novel use of antibiotic-impregnated calcium sulfate beads in five cases of cranioplasty at our institution.

## Case series

### Methods

**Figure 1:**
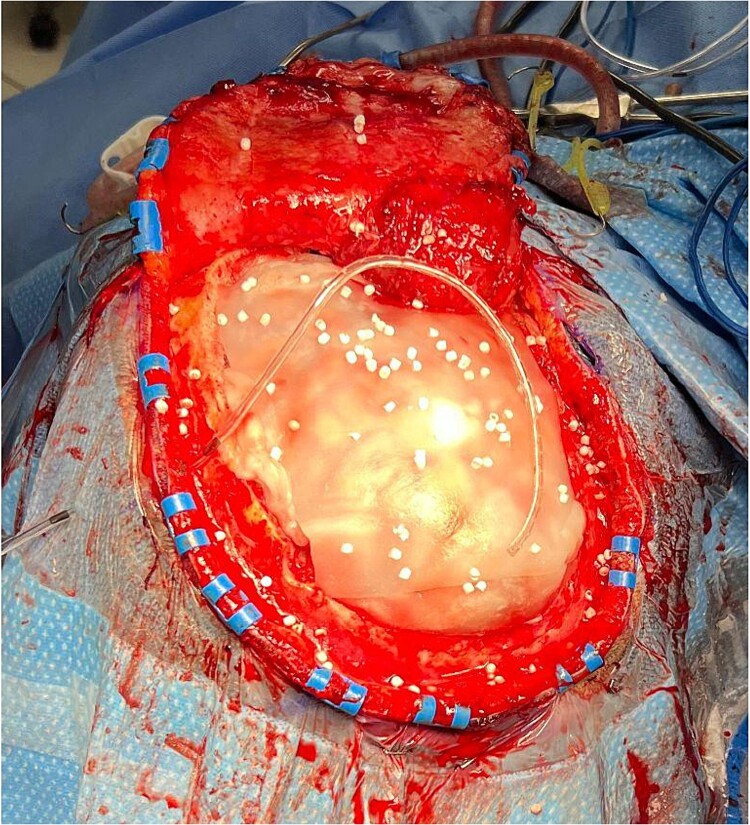
Antibiotic-impregnated dissolvable synthetic calcium sulfate beads (Stimulan Rapid Cure) in a patient undergoing cranioplasty.

A single center retrospective chart review was performed and patients were identified by those receiving off-label use of extradural absorbable antibiotic-impregnated synthetic calcium sulfate beads (STIMULANⓇ Rapid Cure, Biocomposites Ltd, Wilmington, NC, USA) following cranioplasty (Fig. 1) performed by a single surgeon at the University of Oklahoma Health Sciences Center between July 13, 2023 to November 9, 2023. All beads were impregnated with 500 g of vancomycin and 3 mL of tobramycin and ranged from small (3 mm) to medium (4.8 mm) in size. Sex, age, indication for procedure, procedure type, bead size, complications, and time to complication were obtained from the electronic medical record (EMR). Time to follow-up was determined from time of procedure to time of most recent documented hospital or clinic visit via EMR review on May 22, 2024.

## Results

Five patients were included in this study. Two were male and three were female with a mean age of 49.2 ± 10.9 years (range, 33–66). Of our cohort, four patients experienced wound healing as expected with no complications or side effects related to the use of STIMULANⓇ beads. One patient’s clinical course was complicated by pseudomeningocele with superficial wound infection occurring 74 days following cranioplasty. Of note, this patient had suffered an avulsion injury and subgaleal hematoma of the scalp ipsilateral to the cranial incision. No further complications were observed in this cohort. Average time to follow-up was 8.8 ± 1.6 months (range, 6.5–10.4). Indication for cranioplasty and additional information regarding procedure type can be found in Table 1.

**Table 1:** Summary of patients who underwent cranioplasty with the use of antibiotic-impregnated dissolvable synthetic calcium sulfate beads (STIMULANⓇ Rapid Cure).

**Patient number**	**Sex**	**Age**	**Indication for cranioplasty**	**Procedure**	**Bead size**	**Complications**	**Time to follow-up (months)**	**Time to complication (days)**
1	F	33	Wound dehiscence of prior cranioplasty	Right cranioplasty with PEEK implant, fasciocutaneous advancement flap	4.8 mm	None	10.4	N/A
2	M	67	Acute ischemic right MCA stroke	Right hemicraniectomy with cranioplasty	4.8 mm	None	8.9	N/A
3	F	43	Acquired skull defect	Right hemicraniectomy with cranioplasty	4.8 mm	Pseudomeningocele, surgical site infection	9.9	74
4	M	52	CVA due to thrombosis of cerebral artery	Left hemicraniectomy, cranioplasty	3 mm	None	7.6	N/A
5	F	52	SDH	Left hemicraniectomy, evacuation of hematoma, cranioplasty	4.8 mm	None	6.5	N/A

Abbreviations: M = male, F = female, PEEK = polyetheretherketone, N/A = not applicable, MCA = middle cerebral artery, CVA = cerebrovascular accident, SDH = subdural hematoma.

## Discussion

In the setting of cranioplasty, it is likely that extradural administration of antibiotic-impregnated calcium sulfate beads (STIMULANⓇ) provides a safe and effective method of preventing infection in the perioperative period. The majority of patients were complication-free at time of EMR review, with one patient experiencing pseudomeningocele with superficial wound infection 74 days following surgery (Table 1). However, this patient originally presented with a skull avulsion, which may predispose to wound infection or subgaleal collection secondary to incompetent scalp vasculature. Moreover, time to infection following cranioplasty (autologous or otherwise) has been reported anywhere from 35 to 40 days postoperatively [17, 18]; thus, it is inconclusive if the observed complication occurred due to superficial wound contamination from skin flora, compromised scalp vasculature, or inadequate infection prophylaxis at time of cranioplasty. As such, this complication could not be definitively attributed to extradural STIMULANⓇ administration.

To our knowledge, this is the first report of the use of antibiotic-impregnated calcium sulfate beads used off-label with the aim of preventing infection following cranioplasty. Previous efforts to prevent perioperative infection in the setting of cranioplasty have tailored prophylactic antibiotics to each patient’s colonizing flora, which has been significantly associated with decreased infection rates when compared with conventional prophylactic antibiotic regimens [19]. However, the use of Stimulan beads has most notably been adopted in the field of orthopedic surgery, chiefly in the setting of arthroplasty, bone/joint infection, or acquired osteomyelitis [4–6, 11, 13–15, 20–22]. Furthermore, the efficacy and adaptability of this technology has led to its adoption in fields such as plastic and general surgery, as there have been documented cases of the use of STIMULANⓇ (as prophylaxis or salvage therapy) in both breast implantation and hernia repair [23–25].

Moreover, Kenna and colleagues exemplified the utility of this technology in breast reconstruction surgery, as tissue expander loss secondary to infection was 1.5% when using STIMULANⓇ prophylactically compared with 11.9% when using intravenous antibiotics alone [24]. What is more, Sherif *et al.* [23] demonstrated the ability of STIMULANⓇ to treat active infection in breast implantation, with successful implantation salvage observed in 75% of patients receiving the beads; further groups have demonstrated the resolution of active infection in long bones following STIMULANⓇ use [13, 20, 21], with infection remission rates being as high as 76.7% in cases of chronic osteomyelitis [14]. In addition, STIMULANⓇ has been observed to encourage osteoconduction and bone regeneration following partial fibulectomy in pediatric patients [12], which exemplifies the broad utility of antibiotic-impregnated calcium sulfate beads.

Although STIMULANⓇ Rapid Cure technology demonstrates profound promise in minimizing perioperative infection rates, notable complications have been reported in the literature. Due to the chemical profile and bioavailable nature of the beads, systemic hypercalcemia has been observed, with rare cases going on to develop dystrophic calcification or acute kidney injury [5, 22]. However, hypercalcemia secondary to STIMULANⓇ use is generally transient with no indication for medical intervention, and no patients in the current study experienced changes in plasma calcium following Stimulan bead administration.

This work is subject to a variety of limitations and confounding bias as a consequence of limited sample size and follow-up time. Future directions should include randomized prospective cohort studies to determine the true postsurgical infection rates of those undergoing cranioplasty who receive traditional infection prophylaxis compared with antibiotic-impregnated calcium sulfate beads. It is our hope that these preliminary data demonstrate the potential for diverse utility of STIMULANⓇ beads in neurosurgical procedures, albeit within the limitations of the small sample size and minimal follow-up period of the current work.

We report preliminary data regarding the novel use of synthetic absorbable antibiotic-impregnated calcium sulfate beads (STIMULANⓇ) in cranioplasty as applied in an extradural manner prior to closing the skull. Furthermore, the majority of patients treated with STIMULANⓇ were complication-free at follow-up, which exemplifies the need for judicious selection of postoperative infection prevention modalities, as those with compromised scalp vasculature may be predisposed to the development of subgaleal hemorrhage and/or wound infection. Although a larger sample size is necessary to determine a true complication profile, these preliminary data encourage further study of STIMULANⓇ use in neurosurgical procedures.
