# Correction to: Safety and efficacy of antibiotic-impregnated absorbable calcium sulfate beads (Stimulan) in cranioplasty

**DOI:** 10.1093/jscr/rjaf039

**Published:** 2025-02-10

This is a correction to: Alexander R Evans, Marianne E Kimmell, Abdurrahman F Kharbat, Hakeem J Shakir, Safety and efficacy of antibiotic-impregnated absorbable calcium sulfate beads (Stimulan) in cranioplasty, *Journal of Surgical Case Reports*, Volume 2024, Issue 7, July 2024, rjae468, https://doi.org/10.1093/jscr/rjae468

A correction has been made to the second author's last name in the article. This now reads: “Marianne E Kimmell”.

